# Retinoblastoma protein promotes oxidative phosphorylation through upregulation of glycolytic genes in oncogene-induced senescent cells

**DOI:** 10.1111/acel.12351

**Published:** 2015-05-25

**Authors:** Shin-ichiro Takebayashi, Hiroshi Tanaka, Shinjiro Hino, Yuko Nakatsu, Tomoka Igata, Akihisa Sakamoto, Masashi Narita, Mitsuyoshi Nakao

**Affiliations:** 1 Department of Medical Cell Biology, Institute of Molecular Embryology and Genetics, Kumamoto UniversityKumamoto, Japan; 2 Program for Leading Graduate Schools ‘HIGO (Health life science: Interdisciplinary and Glocal Oriented) Program’, Kumamoto UniversityKumamoto, Japan; 3 Cancer Research UK Cambridge Institute, University of Cambridge, Li Ka Shing CentreCambridge, UK; 4 Core Research for Evolutional Science and Technology (CREST), Japan Science and Technology AgencyTokyo, Japan

**Keywords:** gene regulation, glycolysis, metabolic remodeling, oncogene-induced senescence, oxidative phosphorylation, retinoblastoma protein

## Abstract

Metabolism is closely linked with cellular state and biological processes, but the mechanisms controlling metabolic properties in different contexts remain unclear. Cellular senescence is an irreversible growth arrest induced by various stresses, which exhibits active secretory and metabolic phenotypes. Here, we show that retinoblastoma protein (RB) plays a critical role in promoting the metabolic flow by activating both glycolysis and mitochondrial oxidative phosphorylation (OXPHOS) in cells that have undergone oncogene-induced senescence (OIS). A combination of real-time metabolic monitoring, and metabolome and gene expression analyses showed that OIS-induced fibroblasts developed an accelerated metabolic flow. The loss of RB downregulated a series of glycolytic genes and simultaneously reduced metabolites produced from the glycolytic pathway, indicating that RB upregulates glycolytic genes in OIS cells. Importantly, both mitochondrial OXPHOS and glycolytic activities were abolished in RB-depleted or downstream glycolytic enzyme-depleted OIS cells, suggesting that RB-mediated glycolytic activation induces a metabolic flux into the OXPHOS pathway. Collectively, our findings reveal that RB essentially functions in metabolic remodeling and the maintenance of the active energy production in OIS cells.

## Introduction

Cellular senescence is a state of essentially irreversible growth arrest that is induced by various stresses, such as shortened telomeres, DNA damage, and oncogene activation. It is thought to function as an exclusion of unnecessary cells or as an anticancer mechanism. Senescent cells usually exhibit morphological features including an enlarged size, flattened shape, increased β-galactosidase activity, a senescence-associated secretory phenotype, and the formation of senescence-associated heterochromatin foci (SAHF) (Campisi & d’Adda di Fagagna, 2007; Kuilman *et al*., 2010; Rodier & Campisi, 2011; Lopez-Otin *et al*., 2013; Munoz-Espin & Serrano, 2014; Perez-Mancera *et al*., 2014; Salama *et al*., 2014). Senescent cells also possess some dynamic functions that together make up the metabolically active phenotype.

Several signaling pathways that activate the senescence program have been identified *in vitro*, and a deficiency of these pathways is often found in tumors *in vivo* (Collado & Serrano, 2010), suggesting that bypassing cellular senescence could lead to tumorigenesis. Among such pathways, the retinoblastoma protein (RB) has been shown to play a pivotal role in the transcriptional repression of cell cycle genes in both oncogene-induced senescence (OIS) and replicative senescence (RS) (Ben-Porath & Weinberg, 2005). In senescent cells, RB represses E2F target genes including cell cycle regulators (Narita *et al*., 2003), and this repression is partly caused by the RB-mediated recruitment of transcriptional repressors such as histone deacetylases to the regulatory sites of its target genes (Brehm *et al*., 1998; Luo *et al*., 1998; Magnaghi-Jaulin *et al*., 1998). Interestingly, RB is required for SAHF formation through cooperation with histone chaperone proteins HIRA and antisilencing function 1A (Narita *et al*., 2003; Ye *et al*., 2007). RB is also known to act as a transcriptional activator in some contexts (Talluri & Dick, 2012), although it remains unclear whether RB-mediated transcriptional activation contributes to senescent phenotypes. Thus, RB is likely to orchestrate several senescence-associated phenomena and to contribute to the stable maintenance of the senescence program.

Cellular metabolism produces energy and the materials required for cell structure and function, and its regulation is necessary to meet the energy demands of the cellular state. To generate ATP as a cellular fuel, normal cells mainly use OXPHOS in the presence of oxygen and anaerobic glycolysis in its absence. However, proliferative and cancer cells typically show a bias toward aerobic glycolysis even in the presence of oxygen because of their high energy demand for rapid growth. Recently, the metabolic profiling of mouse embryonic stem cells (ESCs), mouse epiblast stem cells (EpiSC), and human embryonic stem cells (hESCs) found that EpiSCs/hESCs possess higher glycolytic and lower OXPHOS activities compared with ESCs (Zhou *et al*., 2012). Regulation of a set of mitochondrial IV *COX* genes was thought to be involved in this phenomenon. Human-induced pluripotent stem cells also displayed higher glycolytic rates relative to the original somatic cells, similar to hESCs (Varum *et al*., 2011). Although it is still uncertain whether metabolic remodeling is a cause or consequence of cellular reprogramming, these studies clearly indicate that metabolic pathways can change depending on cellular states.

Recent studies have shown that cells undergoing OIS have higher OXPHOS activities compared with growing cells (Quijano *et al*., 2012; Dorr *et al*., 2013; Kaplon *et al*., 2013). However, the mechanisms determining the OIS-associated OXPHOS activation are poorly understood. RB is activated during cellular senescence induction and plays crucial roles in senescence-associated phenotypes (Narita *et al*., 2003; Ben-Porath & Weinberg, 2005). Given that RB has been shown to modulate mitochondrial activity through regulating metabolic gene expression (Takahashi *et al*., 2012), we examined the possibility that RB is involved in the OIS-associated OXPHOS activation in this study. Using oncogenic Ras*-*driven senescence in human fibroblasts, we show that RB is involved in increasing both glycolysis and OXPHOS activities in OIS cells. A combination of live metabolic monitoring, and metabolome and gene expression analyses revealed that the RB-dependent upregulation of metabolic genes at the RNA levels promotes glycolysis and subsequent OXPHOS activities. Our findings demonstrate a novel metabolic role for RB in OIS cells, suggesting that RB is a key molecule linking metabolic remodeling and cell cycle control in the senescent program.

## Results

### RB regulates metabolic remodeling during the induction of senescence

**Fig 1:**
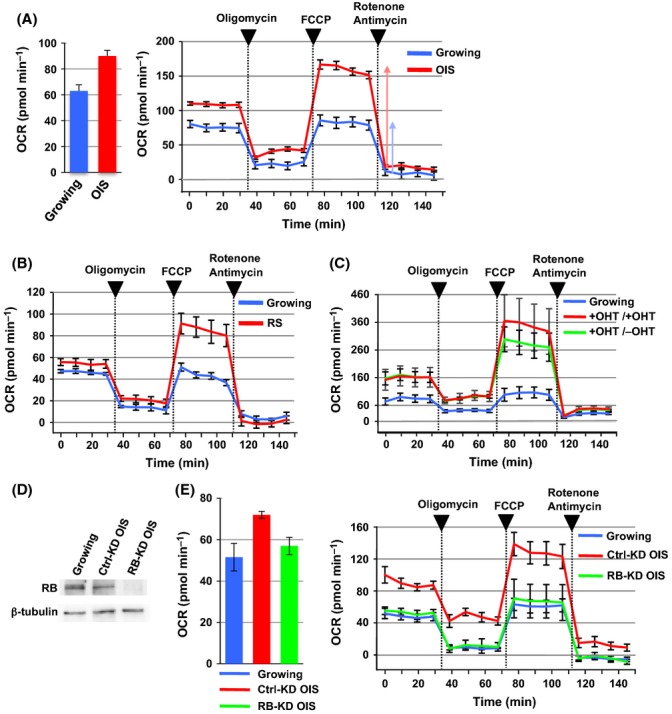
RB is required for increased mitochondrial OXPHOS activity in OIS cells. (A) Oxygen consumption rate (OCR) in growing and oncogene-induced senescence (OIS) cells. OIS was induced in IMR90 cells expressing oncogenic Ras (H-*ras*V12) following treatment with 4-hydroxytamoxifen (4-OHT) for 6 days. OCR was measured as the mitochondrial OXPHOS activity, using the extracellular flux analyzer (normalized to cell number). Respiratory chain inhibitors were added to the culture at the indicated time points. Values are means ± SE of at least three technical replicates at each time point (see Experimental procedures for details). Data are representative of two independent experiments. Basal OCRs are shown by subtracting the rotenone-/antimycin-treated values from the initial values (prior to oligomycin addition) (left). Vertical arrows indicate the maximum OXPHOS capacity determined as the difference between the FCCP- and rotenone-/antimycin-treated OCR values. (B) OCR in growing and replicative senescence (RS) cells. (C) The effect of oncogenic Ras removal on OCR in established OIS cells. OIS cells were established in the presence of 4-OHT for 6 days and further cultured in the presence (+OHT/+OHT) or absence of 4-OHT (+OHT/−OHT) for 4 days (Fig. S1A). (D) RNA interference-mediated knockdown of RB (RB-KD). Control siRNA or siRNAs against *RB1* were introduced to OIS-inducing IMR90 cells (Fig. S1B). (E) The effect of RB-KD on OCR in OIS cells.

To characterize senescence-associated metabolic changes, we used an extracellular flux analyzer to assess the oxygen consumption rate (OCR), which mainly reflects the levels of mitochondrial OXPHOS activity, in human diploid fibroblasts IMR90 (growing) and the same cells undergoing OIS. OIS cells were prepared by 4-hydroxytamoxifen (4-OHT)-inducible Ras (H-*ras*V12) for 6 days as described previously (Hirosue *et al*., 2012). OIS cells showed significantly higher basal OCRs compared with control growing cells (Fig.1A, left), in agreement with previous reports (Quijano *et al*., 2012; Dorr *et al*., 2013; Kaplon *et al*., 2013). Furthermore, as indicated by the vertical arrows in Fig.1A, the maximum OXPHOS capacity was found to be dramatically increased in OIS cells following the addition of carbonyl cyanide-*p*-trifluoromethoxyphenylhydrazone (FCCP), the proton gradient discharger (Fig.1A, right).

A comparable increase in the maximum OXPHOS capacity was found in RS cells that were prepared by repeated proliferation and free from Ras activation (Fig.1B), indicating that senescent cells possess an increased mitochondrial OXPHOS activity. To test the involvement of the oncogenic signal, we removed Ras expression from already established OIS cells. Interestingly, OIS cells maintained higher levels of OXPHOS capacity even after a withdrawal of 4-OHT (Fig.1C and Fig. S1A, +OHT/−OHT). These results suggest that increased OXPHOS capacity in OIS cells does not result from a direct action of the oncogene.

Because RB has a significant function in cellular senescence and plays crucial roles in senescence-associated phenotypes (Narita *et al*., 2003; Ben-Porath & Weinberg, 2005), we next examined whether it is involved in the mitochondrial OXPHOS capacity of OIS cells. To deplete the RB protein, cells were treated with a mixture of three small interfering RNAs (siRNAs) against *RB1* 2 days after Ras induction (Fig.1D and Fig. S1B). RB knockdown of OIS (RB-KD OIS) reactivated genes encoding cell cycle progression proteins (*MCM3* and *MCM5*), and inflammatory cytokines (*IL6* and *IL8*) to a lesser extent, and moderately diminished the population of SAHF-positive cells (Fig. S1C,D). DNA replication, as measured by 5-ethynyl-2′-deoxyuridine (EdU) incorporation, was partially recovered in RB-KD OIS cells, probably from the activation of certain checkpoint pathways (Fig. S1E) (Chicas *et al*., 2010).

As expected, RB depletion by a mixture or individual siRNAs canceled the increase in OCR upon oncogene activation (Fig.1E and Fig. S2A). To further clarify the RB function, RB-KD was performed in already established OIS cells (Fig. S2B). Under this RB-KD condition, the increased mitochondrial OXPHOS was also reversed, suggesting that the effect of RB-KD on OCR in OIS cells is not a consequence of incomplete senescence induction. Moreover, RB-KD similarly reversed the activation of the OXPHOS capacity in RS cells (Fig. S2C). Taken together, we concluded that RB is required for the activation of mitochondrial OXPHOS activity in senescent cells.

### Metabolome profiling reveals the RB-dependent dynamics of cellular metabolites

**Fig 2:**
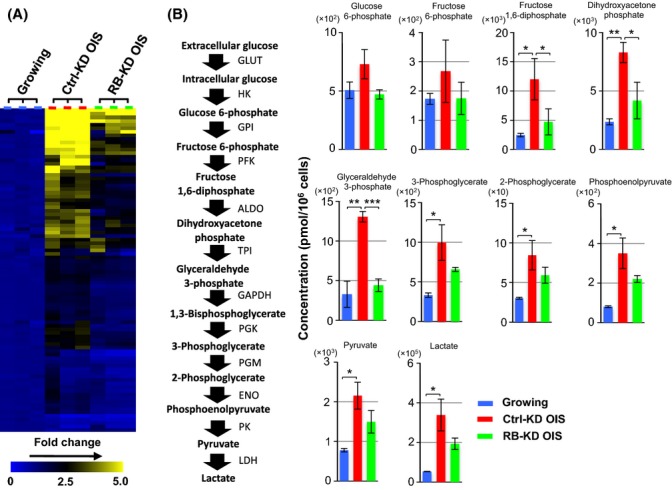
RB-dependent metabolic remodeling in OIS cells. (A) Heat map depicts the relative abundance of 103 metabolites. Metabolome (CE-TOFMS/QqQMS) analyses were performed using three replicates for each cell condition. The color coding indicates the fold change. (B) Metabolites involved in the glycolytic pathway. Diagram of glycolytic pathway is shown (left). Statistical analysis was performed using Welch’s *t*-test (*P <* *0.05; **P <* *0.01; ***P <* *0.001). 1,3-bisphosphoglycerate was not detected in this analysis. Abbreviations: GLUT, glucose transporter; HK, hexokinase; GPI, glucose-6-phosphate isomerase; PFK, phosphofructokinase; ALDO, aldolase; TPI, triose phosphate isomerase; GAPDH, glyceraldehyde-3-phosphate dehydrogenase; PGK, phosphoglycerate kinase; PGM, phosphoglycerate mutase; ENO, enolase; PK, pyruvate kinase; LDH, lactate dehydrogenase.

To further understand RB-mediated metabolic remodeling in cellular senescence, we next carried out a metabolome analysis of each of three independent samples from growing IMR90, Ctrl-KD OIS, and RB-KD OIS cells (Fig.2A and Table S1). We used capillary electrophoresis-time of flight mass spectrometry/triple quadrupole mass spectrometry (CE-TOFMS/QqQMS) to identify 103 metabolites in cellular metabolic pathways. Consistent with previous reports (Quijano *et al*., 2012; Kaplon *et al*., 2013), we detected increased levels of metabolites from glycolysis, the pentose phosphate pathway, and the tricarboxylic acid (TCA) cycle in Ctrl-KD OIS cells (Fig.2B and Fig. S3). The increase in ATP in Ctrl-KD OIS cells is highly compatible with the activated metabolic flow (Fig. S3C). Notably, we found that RB depletion largely attenuated the OIS-associated increase in the levels of these metabolites. Several metabolic intermediates in glycolysis (fructose 1,6-diphosphate, dihydroxyacetone phosphate, and glyceraldehyde 3-phosphate) showed a remarkable reduction in RB-KD OIS cells compared with Ctrl-KD OIS cells. This suggests that RB mediates activation of the glycolytic pathway, which may lead to the increased mitochondrial OXPHOS capacity.

**Fig 3:**
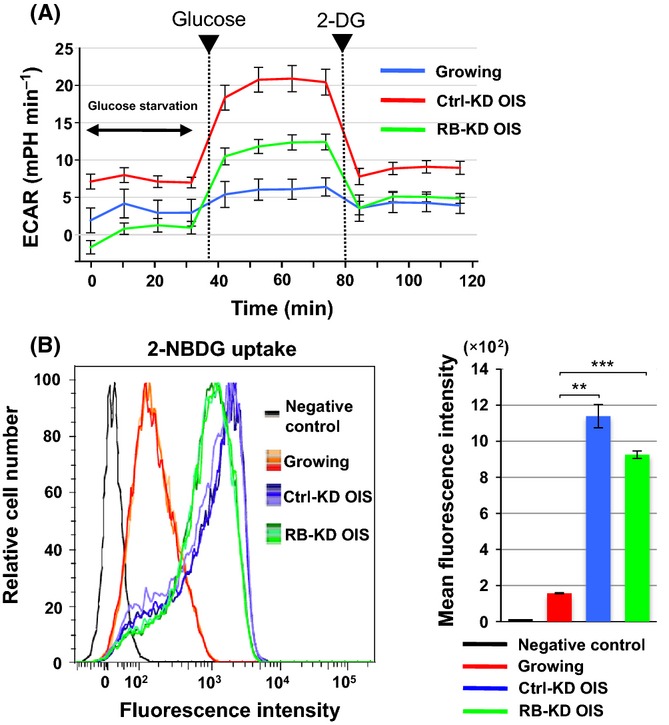
OIS cells enhance RB-dependent glycolytic properties. (A) Extracellular acidification rate (ECAR) in growing and OIS cells. ECAR was measured as the glycolytic activity using the extracellular flux analyzer (normalized to cell number). Real-time monitoring of ECAR was performed under glucose starvation and re-addition (25 mm), completed by adding 2-deoxy-D-glucose (2-DG), a glycolytic inhibitor, at the indicated time points. Values are means ± SE of at least three technical replicates at each time point. Data are representative of two independent experiments. (B) Effect of RB-KD on glucose uptake in OIS cells. Glucose incorporation rate was determined by flow cytometry using 2-NBDG, a fluorescent glucose analog, in growing, Ctrl-KD OIS, and RB-KD OIS cells. Cells that were untreated with 2-NBDG were used as a negative control. From the histograms of biological triplicate samples (left), mean fluorescence intensities were calculated and shown in the right panel (means ± SD). Statistical analysis was performed using Welch’s *t*-test (**P <* *0.01; ***P <* *0.001).

To test RB-dependent glycolytic activation in OIS cells, we further measured the extracellular acidification rate (ECAR) under glucose starvation and subsequent addition, an index of glycolytic activity (Fig.3A). The loss of RB in OIS cells decreased the ECAR compared with the OIS control. We then performed a kinetic analysis of glucose uptake, using 2-deoxy-2-[(7-nitro-2,1,3-benzoxadiazol-4-yl)amino]-d-glucose (2-NBDG), a fluorescent glucose analog (Fig.3B). The Ctrl-KD OIS cells showed a marked increase in glucose uptake compared with growing cells, while RB-KD OIS cells showed a similar uptake to Ctrl-KD OIS cells. This suggests that RB regulates the glycolytic pathway rather than glucose uptake.

### RB upregulates glycolytic genes at the RNA levels in OIS cells

**Fig 4:**
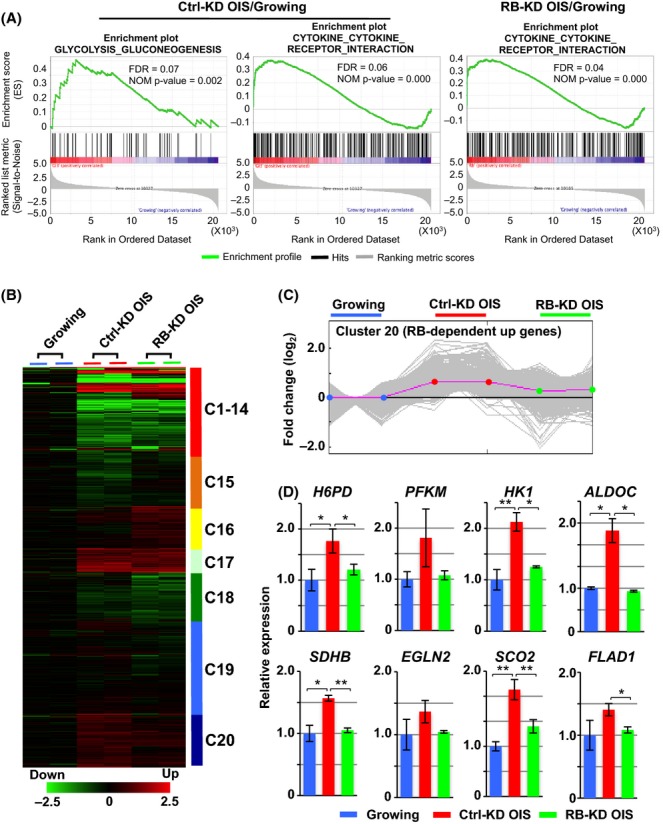
RB mediates the upregulation of a set of glycolytic metabolism genes. (A) Gene set enrichment analysis of upregulated genes in OIS cells. In each panel, nominal *P*-values and false discovery rates (FDRs) are indicated. The ‘glycolysis and gluconeogenesis’ gene set was ranked as significantly upregulated in Ctrl-KD OIS but not RB-KD OIS cells. (B) Heat map of gene expression data obtained from growing, Ctrl-KD OIS, and RB-KD OIS cells (*n* = 2 for each condition). The color coding indicates fold change in expression levels (log2-transformed). Genes were categorized into 20 clusters based on K-means clustering. (C) Expression patterns of 1748 genes in Cluster 20. The pink line represents the average of Cluster 20 genes. The *y*-axis is in log2 scale. (D) RT–PCR analysis of metabolic genes in Cluster 20 in growing, Ctrl-KD OIS, and RB-KD OIS cells. Data are shown as means ± SD of three independent experiments. Statistical analysis was performed using Welch’s *t*-test (*P <* *0.05; **P <* *0.01). Abbreviations: H6PD, hexose-6-phosphate dehydrogenase; PFKM, phosphofructokinase, muscle; HK1, hexokinase 1; ALDOC, aldolase C, fructose-bisphosphate; SDHB, succinate dehydrogenase complex, subunit B; EGLN2, Egl-9 family hypoxia-inducible factor 2; SCO2, SCO2 cytochrome c oxidase assembly protein; FLAD1, flavin adenine dinucleotide synthetase 1.

To examine how RB regulates metabolic gene expression, we performed genome-wide expression microarray analysis in growing, Ctrl-KD OIS, and RB-KD OIS cells. Gene set enrichment analysis (GSEA) using Kyoto Encyclopedia of Genes and Genomes gene sets was performed to identify RB-dependent biological pathways (Fig.4A and Table S2). We found that the ‘glycolysis and gluconeogenesis’ gene set was uniquely upregulated in Ctrl-KD OIS but not RB-KD OIS cells, indicating that activation of the glucose metabolism pathway is RB-dependent in OIS cells. By contrast, some gene sets, such as ‘cytokine and cytokine receptor interaction’, were upregulated in both Ctrl-KD OIS and RB-KD OIS cells. This suggests that activation of the cytokine pathway is RB-independent.

To further elucidate the RB-mediated transcriptional regulation of metabolic genes, we performed a K-means clustering of the 14 042 genes on the microarrays. This analysis revealed 20 gene clusters with distinct patterns of up- and downregulation in each cell state (Fig.4B). Cluster 20 contained a set of genes that were upregulated in Ctrl-KD OIS cells but downregulated in RB-KD OIS cells (Fig.4C). Based on gene ontology (GO) analysis, 307 of the 1748 genes in this cluster were involved in cellular metabolic processes (Table S3). Reverse transcription (RT)–PCR of glycolytic and metabolic genes validated the microarray results (Fig.4D and Fig. S4). According to previous chromatin immunoprecipitation coupled with deep sequencing (ChIP-seq) data (Chicas *et al*., 2010), RB enrichment occurred in association with 36% (109/307) of the metabolic genes in Cluster 20 (Fig. S5 and Table S4), suggesting that both direct and indirect actions of RB are involved in upregulation of a set of metabolic genes. Promoter sequence analysis of RB target genes identified several potential transcription factors (Table S5), some of which are known to cooperate with RB in transcriptional activation (Talluri & Dick, 2012). For example, SP1 has been shown to physically interact with RB and stimulate dihydrofolate reductase gene expression in CHO-K1 cells (Noe *et al*., 1998). We also identified putative binding sites for CCAAT/enhancer-binding protein beta (C/EBPß), which is known to cooperate with RB in the activation of transcription (Chen *et al*., 1996). Among these genes, phosphofructokinase, muscle (*PFKM*) and aldolase C, fructose-bisphosphate (*ALDOC*) are involved in the production of fructose 1,6-diphosphate and dihydroxyacetone phosphate, respectively, which were significantly reduced by RB-KD (Fig.2B, PFK and ALDO). We re-evaluated the previously published gene expression profiles of human SAOS-2 cells in which RB had been reintroduced into a *RB−/−* background (Lopez-Bigas *et al*., 2008). This data set indicated that the RB reintroduction increased mRNAs of metabolic genes shown in Fig.4D, while it decreased the mRNA levels of cell cycle genes such as *MCM3* and *MCM5* (Fig. S6). This suggests a conserved role of RB in the upregulation of metabolic genes in different cellular contexts.

**Fig 5:**
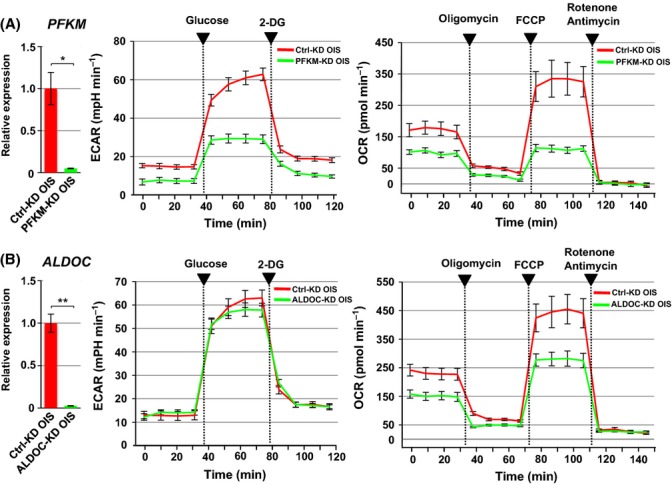
Effect of glycolytic gene knockdown on glycolytic and mitochondrial OXPHOS activities. (A, B) The RB-targeted glycolytic genes *PFKM* and *ALDOC* were depleted using specific siRNAs in OIS cells (left). Data are shown as means ± SD of three independent experiments. ECAR and OCR were measured as described in Figs3A and 1A, respectively (right). Statistical analysis was performed using Welch’s *t*-test (*P <* *0.05; **P <* *0.01).

Finally, we performed a knockdown of these RB target genes. The loss of glycolytic genes (*PFKM* and *ALDOC*) resulted in decrease in glycolytic activity (ECAR) and mitochondrial OXPHOS (OCR) in OIS cells (Fig.5). PFKM-KD reduced glycolytic activity more significantly than ALDOC-KD,possibly resulting in the observed effect on downstream OXPHOS capacity. These data suggest that PFKM rather than ALDOC plays an important role in the metabolic remodeling of OIS cells. In addition, we examined the effect of the gene knockdown on the cellular level of dihydroxyacetone phosphate (DHAP), which is generated by aldolases. RB-KD or ALDOC-KD expectedly showed a decrease in the DHAP content in OIS cells, while PFKM-KD was less effective (Fig. S7). This data may suggest that PFKM has multiple effects on the glycolytic pathway. Indeed, fructose 1,6-diphosphate that is generated by PFKM is known to stimulate formation of highly active tetrameric pyruvate kinase M2 (PKM2), thereby promoting the glycolytic pathway (Mazurek, 2011). Collectively, RB appears to mediate OIS-associated metabolic remodeling at least in part through the upregulation of glycolytic genes. Additionally, throughout this study, we observed that RB was present exclusively in the nuclei but not the cytoplasm of IMR90 and OIS cells (data not shown).

It has been reported that fatty acid oxidation contributes to the increased OXPHOS activity in OIS cells (Quijano *et al*., 2012). Consistently, by treating with etomoxir, a CPT-1 inhibitor, the enhancement of OCR was reduced in OIS cells, compared with the growing (Fig. S8A). Moreover, OCR was increased by the exogenously added free-fatty acid palmitate in Ctrl-KD OIS but not in RB-KD OIS cells (Fig. S8B), suggesting that RB is involved in the regulation of fatty acid oxidation in OIS cells. Thus, our result, together with the previous reports (Quijano *et al*., 2012; Kaplon *et al*., 2013), indicates that both glycolytic and fatty acid oxidation pathways contribute to maintaining the increased OXPHOS capacity in OIS cells.

## Discussion

In this study, we report on the RB mediation of metabolic remodeling in OIS cells. Based on live metabolic monitoring, and metabolome and gene expression analyses, our findings indicate that RB upregulates a series of glycolytic genes, resulting in increased glycolysis and subsequent mitochondrial OXPHOS activities in OIS cells. Thus, OIS cells have activated metabolic flow, in comparison with other cell states.

**Fig 6:**
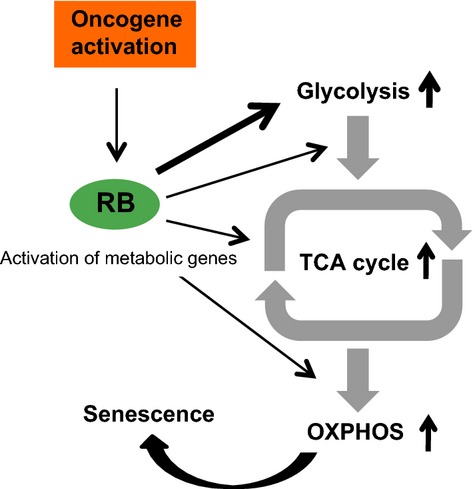
Schematic model for RB-mediated activation of both glycolysis and mitochondrial OXPHOS in OIS cells. In response to oncogenic signals, RB either directly or indirectly upregulates the mRNA levels of target genes such as glycolytic genes. Glycolytic stimulation promotes a metabolite flux into the TCA cycle, leading to OIS-driven mitochondrial OXPHOS activation. Consequently, OIS cells are metabolically activated under growth arrest, compared with normal cells and proliferative cancer cells.

RB has been found to transcriptionally downregulate some metabolic genes as well as cell cycle genes (Hsieh *et al*., 2008; Blanchet *et al*., 2011; Reynolds *et al*., 2014). For example, the glutamine transporter *SLC1A5* was reported to be a direct target of RB transcriptional repression in mouse embryonic fibroblast cells (Reynolds *et al*., 2014). By contrast, our study indicated that RB upregulates glycolytic genes and establishes high activities of glycolytic and mitochondrial OXPHOS pathways in OIS cells (Fig.6). Although the degree of RB-mediated transcriptional activation was found to be relatively weak (Fig.4C,D), the RB-dependent upregulation of multiple metabolic genes may synergistically support a metabolically activated phenotype in OIS cells (Fig.4D and Table S3). Considering that mRNA levels do not always correlate with protein levels, further study may be necessary to address whether the increased mRNAs from metabolic genes are linked to the control of enzymatic activities. A previous study showed that upregulation of the pyruvate dehydrogenase phosphatase 2 gene (*PDP2*) led to enzymatic activation of pyruvate dehydrogenase (PDH), which catalyzes the conversion of pyruvate into acetyl coenzyme A, and thereby promotes a flux from glycolysis to the TCA cycle in OIS cells (Kaplon *et al*., 2013). However, details of the upstream pathways regulating PDP2 levels are currently unknown. Interestingly, the *PDP2* promoter was bound by RB (Table S4), suggesting that RB participates in the regulation of the PDP2–PDH axis. Thus, RB can contribute to the activation of multiple metabolic pathways. Indeed, the OXPHOS gene set also appears to be upregulated in an RB-dependent manner (Table S2.). Our data strongly suggest that RB regulates a set of metabolic genes at the transcriptional level. However, it is also possible that loss of RB resulted in the stabilization of target mRNAs via unknown mechanisms, which could be a cause of the increased mRNA level.

What is the role of high metabolic potentials in OIS cells? Oncogene signaling is thought to induce metabolic remodeling to support the rapid proliferation and survival of transformed cells. For instance, it has been shown that activation of the K-Ras (Kirsten rat sarcoma viral oncogene homolog) induces the transcriptional upregulation of glycolytic genes (Ying *et al*., 2012). Cancer cells typically utilize glycolysis to produce ATP while relying to a lesser extent on OXPHOS, which is known as the Warburg effect (Warburg, 1956; Vander Heiden *et al*., 2009). Considering that OXPHOS produces reactive oxygen species (ROS) (Orrenius *et al*., 2007), a potential source of cellular damage, the suppression of mitochondrial OXPHOS in cancer cells could be advantageous for survival. Thus, the high glycolytic rates induced by oncogenic activation are not directly linked to OXPHOS capacity in cancer cells. Conversely, glycolytic activation in normal cells with an intact mitochondrial function could stimulate downstream OXPHOS and ROS production. Because OIS-inducing cells are likely to maintain intact mitochondrial potentials, the resulting activation of the energy production by RB may have roles in the initiation and maintenance of cellular senescence (Fig.6).

Senescence-associated heterochromatin foci (SAHF) are formed by the spatial repositioning of the genome and is correlated with repressive marks such as trimethylated histone H3 on lysine 9 (H3K9me3) and H3K27me3 (Chandra *et al*., 2012). Although the involvement of epigenetic marks in the OIS state remains poorly understood, alterations in the distribution of histone modifications were previously observed in OIS cells (Chicas *et al*., 2012; Shah *et al*., 2013). Recently, there has been increasing evidence of cross talk between the cellular metabolism and epigenome through cellular metabolites (Hino *et al*., 2013). This is supported by the fact that many epigenetic modifiers utilize metabolites as cofactors or substrates for their enzymatic reactions. Therefore, the levels of cellular metabolites are likely to influence the activity of modifying enzymes at local and whole genomic regions. Indeed, α-KG, an intermediate of the TCA cycle, is required for oxidative lysine demethylation by the Jumonji C domain-containing histone demethylases. In this study, we found that the observed increase in α-KG was RB-dependent in OIS cells (Fig. S3). Additionally, Jumonji/ARID domain-containing protein 1A (JARID1A) and JARID1B, in cooperation with RB, are required for global H3K4me3 demethylation and the silencing of cell cycle gene expression in OIS cells (Chicas *et al*., 2012). Collectively, RB may therefore coregulate the α-KG increase and H3K4me3 demethylation by JARID1A/1B proteins in the initiation and maintenance of a senescent state.

Finally, we discuss the relationship between cellular metabolism and cell cycle progression in OIS cells. It has been reported that the oscillation of 6-phosphofructo-2-kinase/fructose-2,6-bisphosphatase 3 levels is mediated by targeted degradation by the ubiquitin ligase anaphase-promoting complex/cyclosome and Skp1–Cullin1–F-box complex, together with an increase in lactate production during late G1 phase (Colombo *et al*., 2011; Tudzarova *et al*., 2011), suggesting that the cell cycle condition affects metabolism. On the other hand, our present study showed that RB-KD completely reduced mitochondrial OXPHOS activity to the levels seen in growing cells (Fig.1E) and partially rescued OIS-associated cell cycle arrest (Fig. S1E). Because some metabolic genes are direct targets of RB (Fig. S5 and Table S4), we assumed that OIS-associated metabolic remodeling is not simply a consequence of cell cycle arrest. For future studies, it would be of great interest to determine whether RB-mediated upregulation of metabolic genes occurs in senescent cells *in vivo*. Enzymes involved in cancer-specific metabolism are potential therapeutic targets, and some inhibitors of these enzymes have been successful in clinical trials (Cheong *et al*., 2012). Thus, the RB-mediated metabolic remodeling pathway described here could represent a valuable marker and therapeutic target for the prevention of aging-related diseases.

## Experimental procedures

### Cell culture

IMR90 cells expressing 4-OHT-inducible ER:Ras (H-*ras*V12) were maintained in Dulbecco’s modified Eagle’s minimal essential medium F12 (045-30665, Wako, Osaka, Japan), supplemented with 10% (v/v) heat-inactivated fetal bovine serum (Young *et al*., 2009). To induce OIS, the cells were treated with 100 nm 4-OHT for 6 days as described previously (Hirosue *et al*., 2012). IMR90 cells which arrested their growth after 8–10 weeks of culture were used as RS cells. The following siRNAs were transfected using RNAiMax (Life Technologies, Carlsbad, CA, USA): *siRB1* (Santa Cruz Biotechnology, Santa Cruz, CA, USA, sc-44273A [designated as #1], B [#2], and C [#3]), *siPFKM* (Life Technologies, Silencer Select, 10370), *siALDOC* (Life Technologies, Silencer Select, s1263), and the control siCon (siRNA targeted to luciferase GL3) (Hino *et al*., 2012). For RB-KD, a mixture of three *siRB1* (A, B, and C) was used at a concentration of 10 nm unless otherwise indicated.

### Reverse transcription–PCR

Total cellular RNA was isolated from cells using the RNeasy kit (Qiagen, Valencia, CA, USA) according to the manufacturer’s instructions. cDNA was synthesized with the ReverTra Ace® qPCR RT Master Mix (Toyobo, Osaka, Japan), and quantitative reverse transcription (RT)–PCR performed by the SYBR green method using Thunderbird reagents (Toyobo) and an ABI 7300 Sequence Detector (Applied Biosciences, Foster City, CA, USA). The primer sets used in this study are given in Table S6.

### Microarray analysis

Total cellular RNA from two independent experiments was reverse-transcribed and hybridized to the Affymetrix U133 Plus 2.0 microarray, as described previously (Hino *et al*., 2012). Gene set enrichment analysis was performed using GSEA ver. 2.0 software provided by the Broad Institute of MIT and Harvard (http://www.broadinstitute.org/gsea/) (Subramanian *et al*., 2005). K-means clustering with the Euclidean distance was performed using MeV 4.8.1 (Saeed *et al*., 2003), and GO term analysis was carried out with the DAVID functional annotation tool (Dennis *et al*., 2003). RB enrichment in association with Cluster 20 genes (±2 kb from the transcription start site) was determined using PAVIS (Huang *et al*., 2013), based on previously reported RB ChIP-seq data in OIS IMR90 cells (Chicas *et al*., 2010).

### Immunoblot analysis

Cell lysates with sample buffer (50 mm Tris–HCl, pH 6.8, 2% sodium dodecyl sulfate (SDS), 6% β-mercaptoethanol, 10% glycerol, 0.05% bromophenol blue) were separated by SDS-polyacrylamide gel electrophoresis and transferred to nitrocellulose membranes. Anti-RB rabbit polyclonal antibody (554136, Becton Dickinson, Franklin Lakes, NJ, USA) diluted 1:500 and anti-β-tubulin mouse monoclonal antibody (T4026, Sigma, St. Louis, MO, USA) were used as primary antibodies.

### Metabolome analysis

Metabolome analysis was performed at Human Metabolome Technologies (HMT, Tsuruoka, Japan, http://humanmetabolome.com). In brief, cells (growing IMR90, Ctrl-KD OIS, and RB-KD OIS) were washed with 5% mannitol solution, and cellular metabolites were extracted using methanol containing HMT Internal Standard Solution 1 at room temperature. Metabolome analysis was performed by CE-TOFMS/QqQMS. Metabolite peaks were quantified and normalized according to the cell number.

### Measurement of dihydroxyacetone phosphate

Dihydroxyacetone phosphate content was determined using PicoProbe^TM^ Dihydroxyacetone Phosphate Fluorometric Assay Kit (BioVision, Milpitas, CA, USA), according to the manufacturer’s instructions.

### Assessment of glucose incorporation rate

The fluorescent glucose analog 2-NBDG (Peptide Institute Inc., Osaka, Japan) was used to monitor the glucose incorporation rate. Cells cultured in medium containing 100 μm 2-NBDG for 2 h were collected using the Accumax reagent (Innovative Cell Technologies, San Diego, CA, USA), and fluorescence was measured by fluorescence-activated cell sorting with a Canto flow cytometer (Becton Dickinson).

### Real-time measurement of glycolytic and OXPHOS activities

Real-time monitoring of cellular metabolic activities was performed using an XF24 extracellular flux analyzer (Seahorse Bioscience, North Billerica, MA, USA), according to the manufacturer’s instructions. Control and siRNA-treated cells were cultured on the assay culture plate for 24 h before the assay. Before loading the assay culture plate on to the XF24 extracellular flux analyzer, cells were cultured in unbuffered DMEM (DMEM with 25 mm glucose, 1 mm sodium pyruvate, 2 mm L-glutamate, pH 7.4) and incubated in a non-CO_2_ incubator for 1 h at 37 °C. Maximum OXPHOS capacity was determined as previously reported with some modifications (Hino *et al*., 2012). In brief, during the real-time measurement, inhibitors of respiratory chain components were serially added to the culture: the complex V inhibitor oligomycin (1 μm), the respiratory uncoupler FCCP (1.5 μm), and the complex I/III inhibitors rotenone (1 μm) and antimycin A (1 μm). After each addition, the OCR was measured four times. The addition of FCCP accelerates oxygen consumption to a maximum level, whereas complex I inhibitors completely abolish mitochondrial respiration. Thus, the difference in OCR between FCCP- and rotenone/antimycin-treated states indicates the maximum OXPHOS capacity. To test for glycolytic activities, cells were starved in glucose-free medium for 3 h, after which glucose was reintroduced (25 mm). Cells were then treated with 100 mm 2-DG, a glycolytic inhibitor. Real-time OCR and ECAR data in all the figures are representative of at least two independent biological replicates. Values are means ± SE of at least three technical replicates at each time point. For technical replicates, Ras induction and siRNA treatment were performed using at least three independent cultures for each experiment. Briefly, control OIS and knockdown cells from the same original culture were pooled and plated on three-to-six-well SeaHorse microplate for the measurement. To examine the contribution of fatty acid oxidation to OCR in OIS cells, cells were treated with 100 μm etomoxir, a CPT1 inhibitor. To prepare a fatty acid complex that can be incorporated into cells, palmitate was conjugated with BSA in a 6 to 1 molar ratio as described previously (Zhang *et al*., 2012). Palmitate-BSA conjugate (100 μm) was added to the cells just prior to the OCR measurement (*t* = 0).

## Supporting Information

**Fig. S1** Experimental design and the effect of RB-KD on OIS cells. **Fig. S2** Mitochondrial OXPHOS capacity under indicated experimental conditions. **Fig. S3** Effect of RB-KD on metabolic products in OIS cells. **Fig. S4** Effect of RB-KD using individual siRNAs on metabolic gene expression. **Fig. S5** RB enrichment in association with metabolic genes in Cluster 20. **Fig. S6** Results of metabolic gene expression from the previously published dataset. **Fig. S7** Effect of glycolytic gene knockdown on cellular dihydroxyacetone phosphate content. **Fig. S8** Contribution of fatty acid oxidation on mitochondrial OXPHOS activity in OIS cells.

**Table S1** Summary of metabolite profiling in growing (IMR90), Ctrl-KD OIS, and RB-KD OIS cells.

**Table S2** Gene set enrichment analysis of pathways upregulated in Ctrl-KD OIS and RB-KD OIS, compared with growing IMR90 cells.

**Table S3** List of metabolic genes in Cluster 20.

**Table S4** Summary of RB enrichment in association with metabolic genes in Cluster 20.

**Table S5** Putative transcription factor binding sites in metabolic gene promoters.

**Table S6** Primers used in this study.
