# Supplementary figures and images for: Retinoblastoma protein promotes oxidative phosphorylation through upregulation of glycolytic genes in oncogene-induced senescent cells
Source: Aging Cell. 2015 May 25;14(4):689–97. doi: 10.1111/acel.12351 (PMC4531082; doi:10.1111/acel.12351)

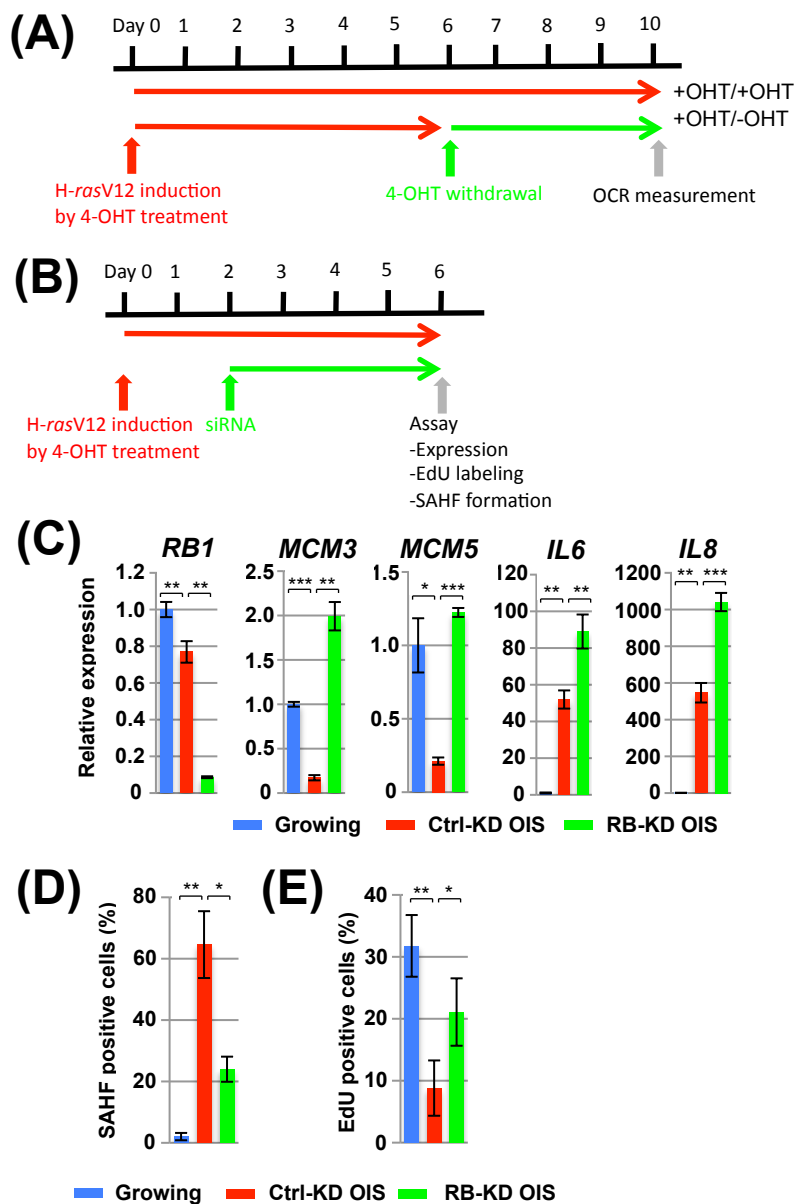

Fig. S1

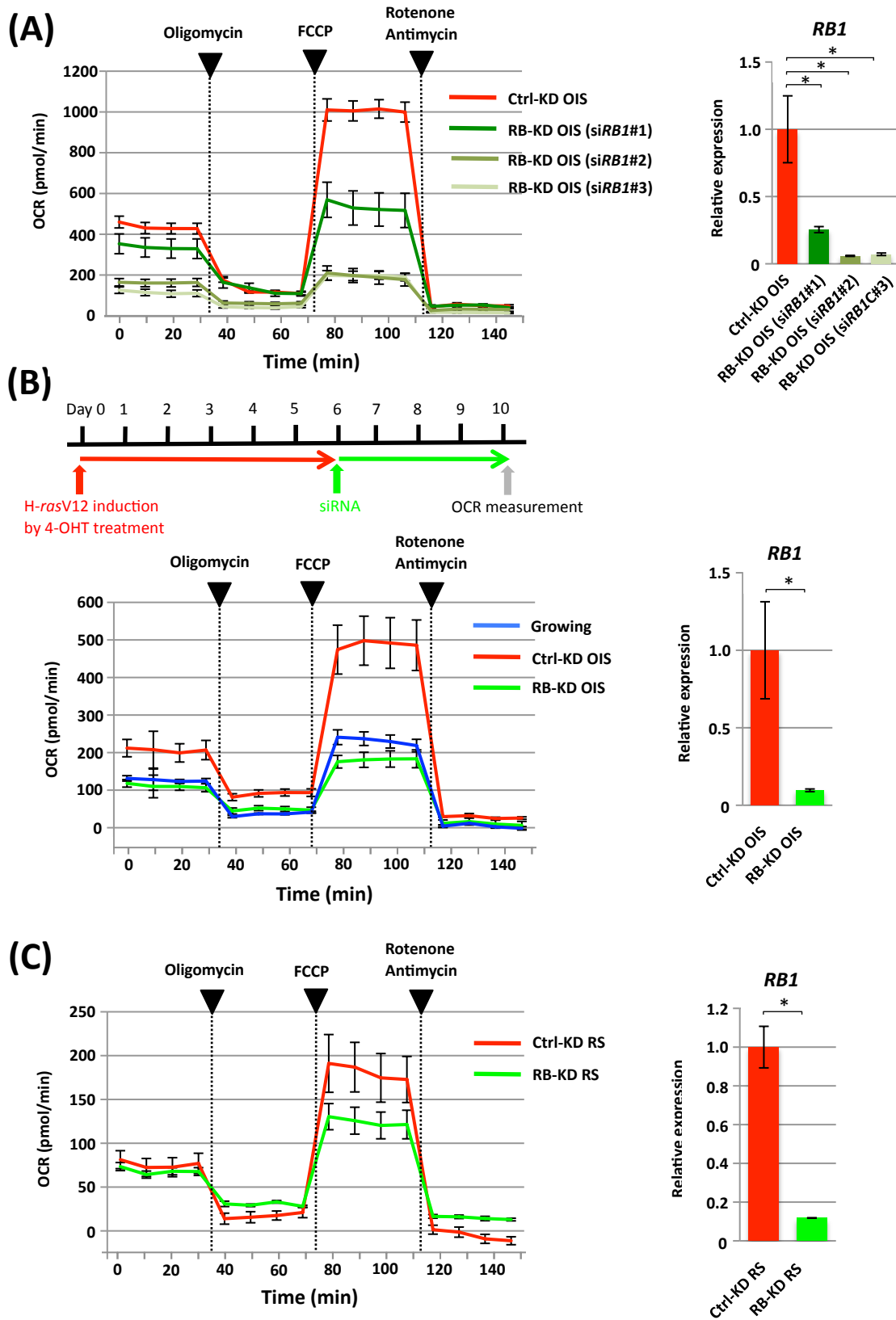

**Fig. S2**

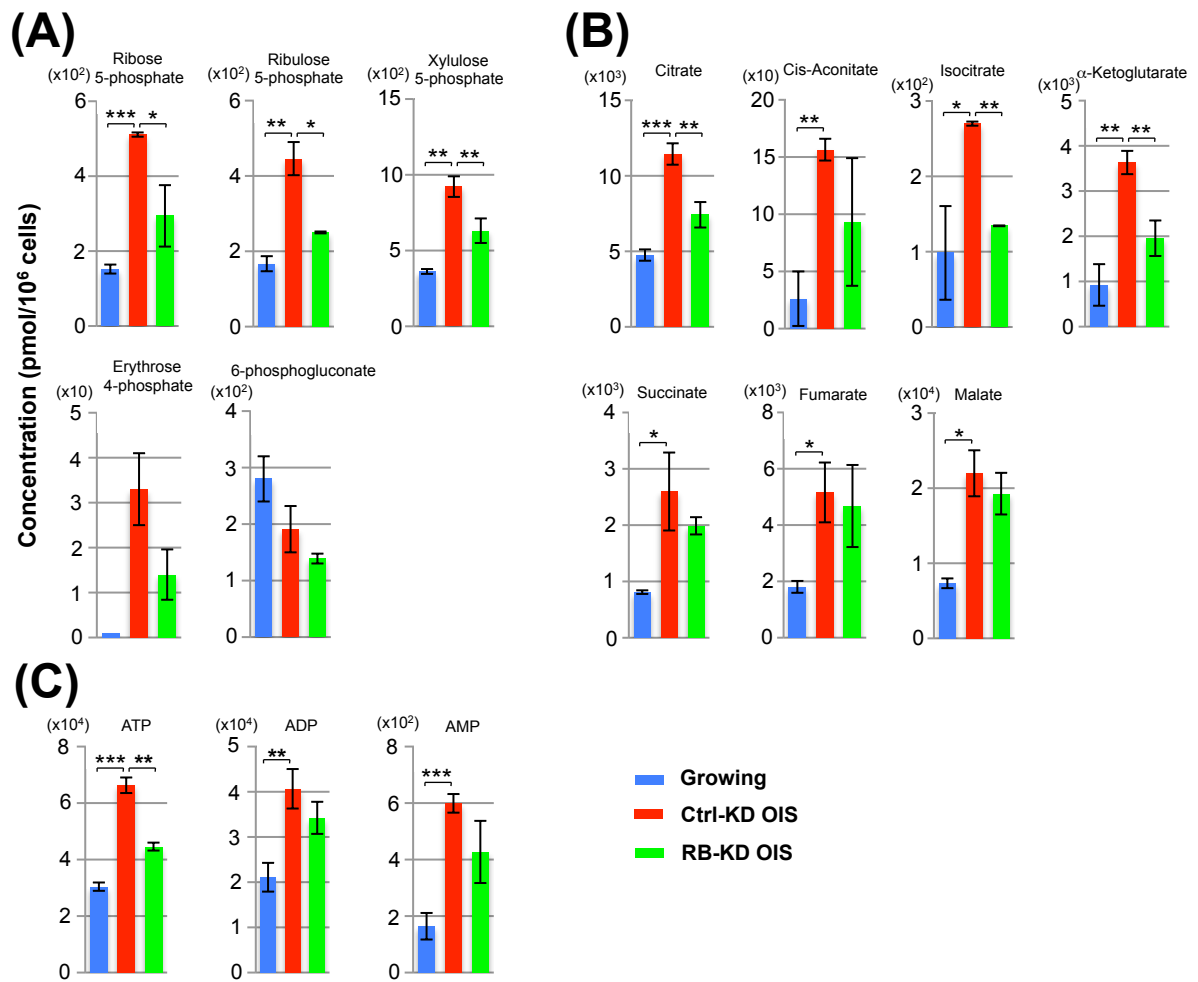

**Fig. S3**

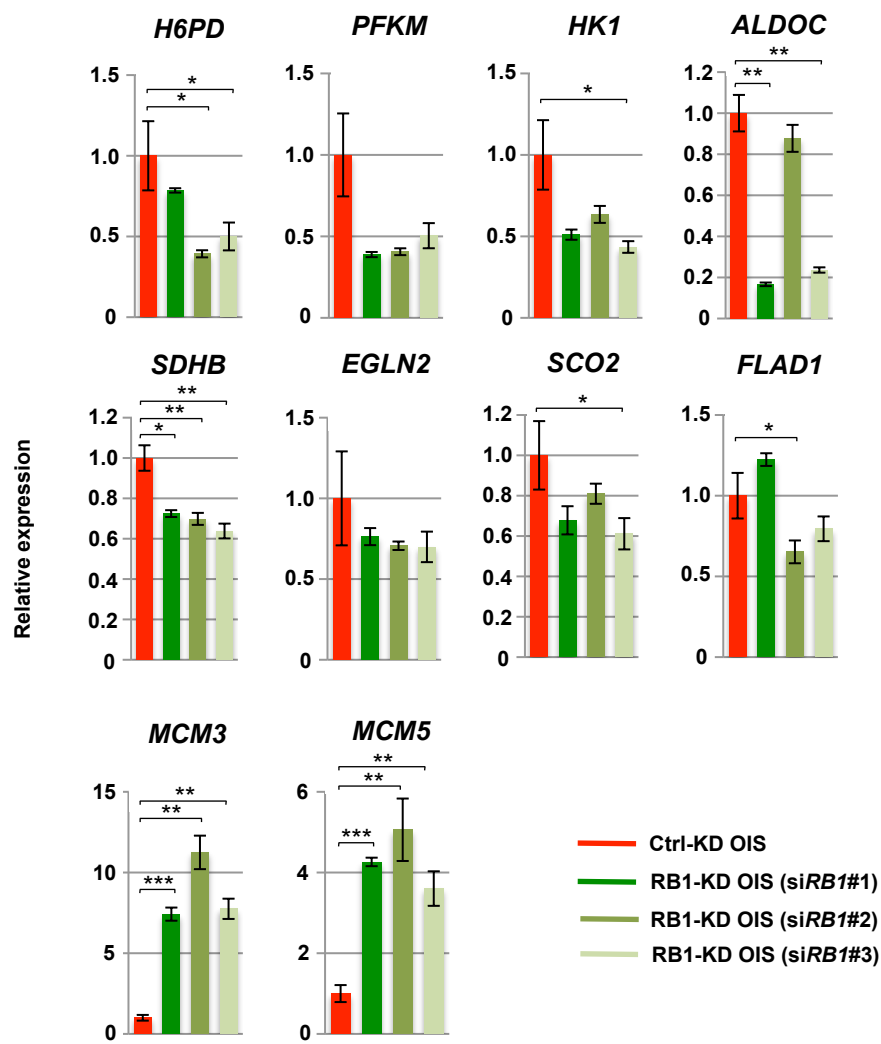

Fig. S4

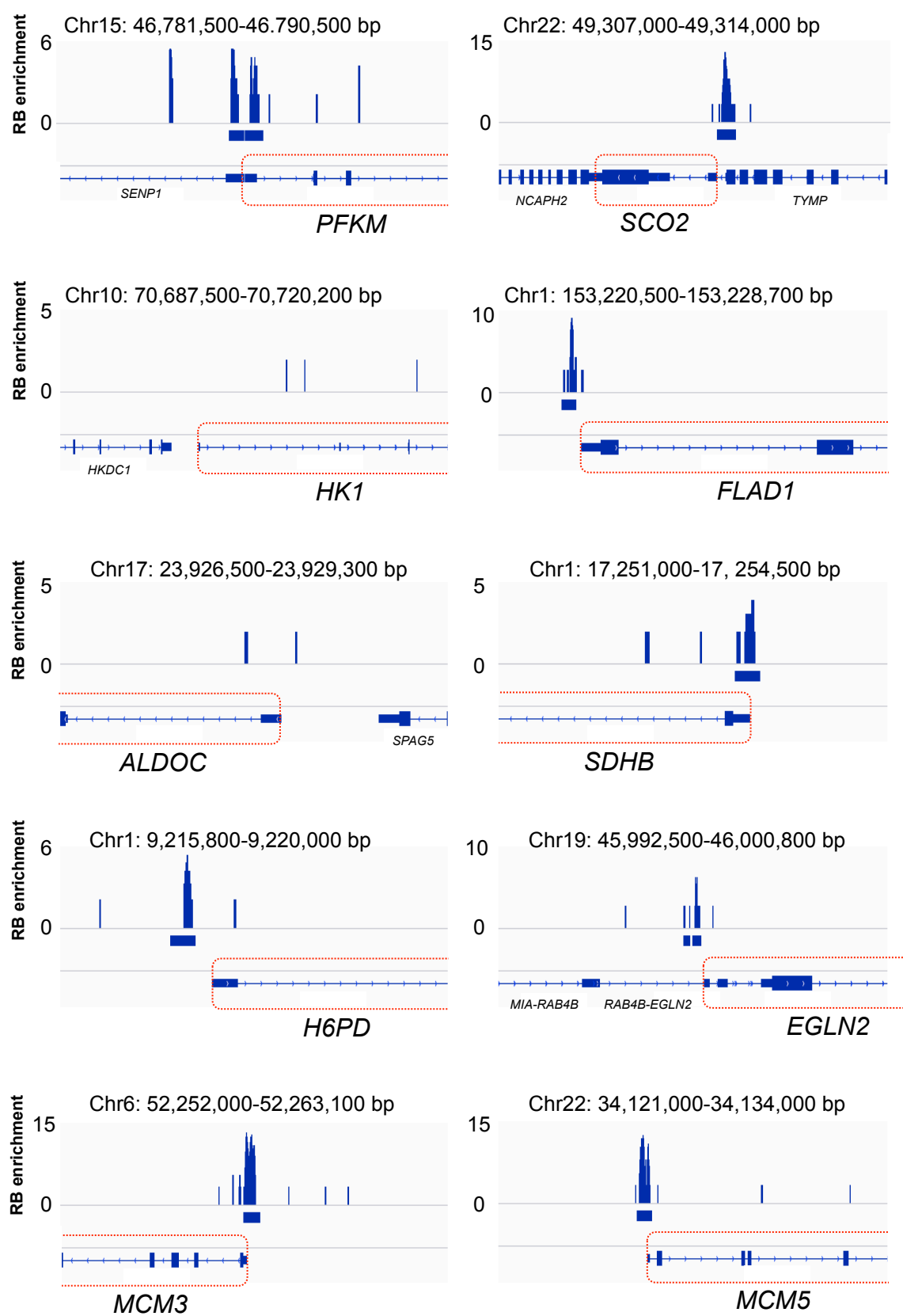

**Fig. S5**

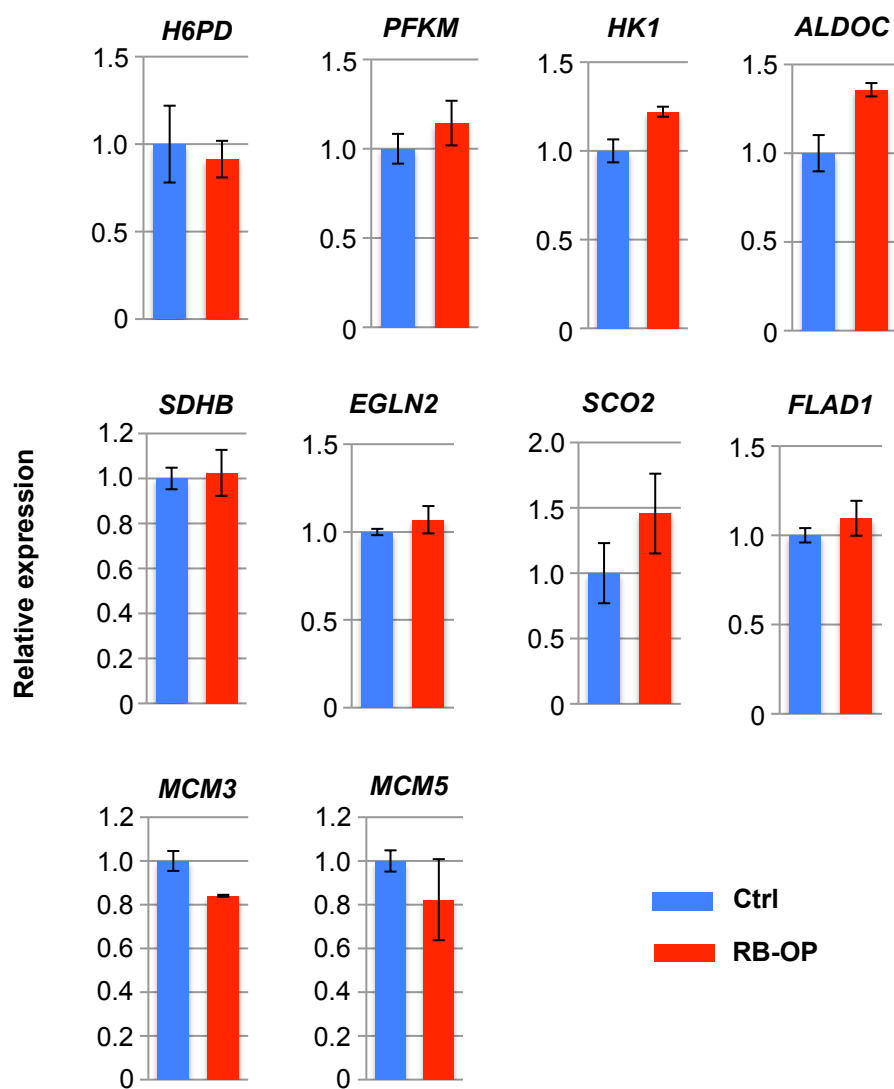

**Fig. S6**

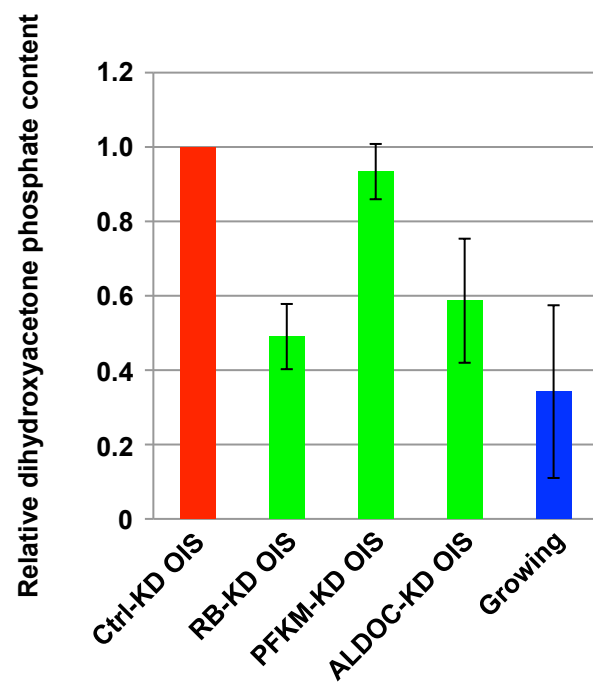

**Fig. S7**

(A)

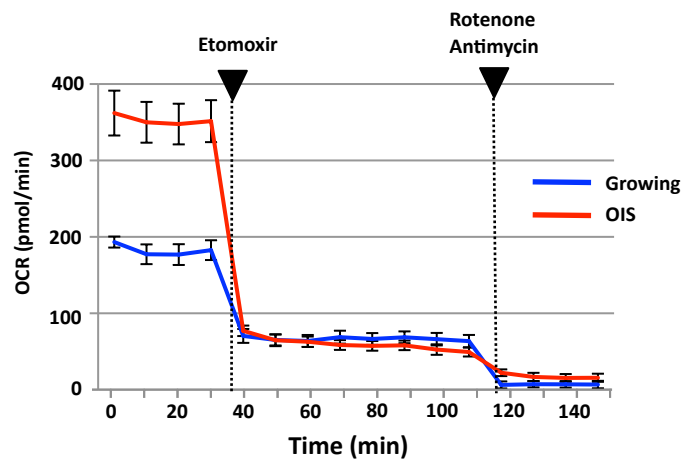

(B)

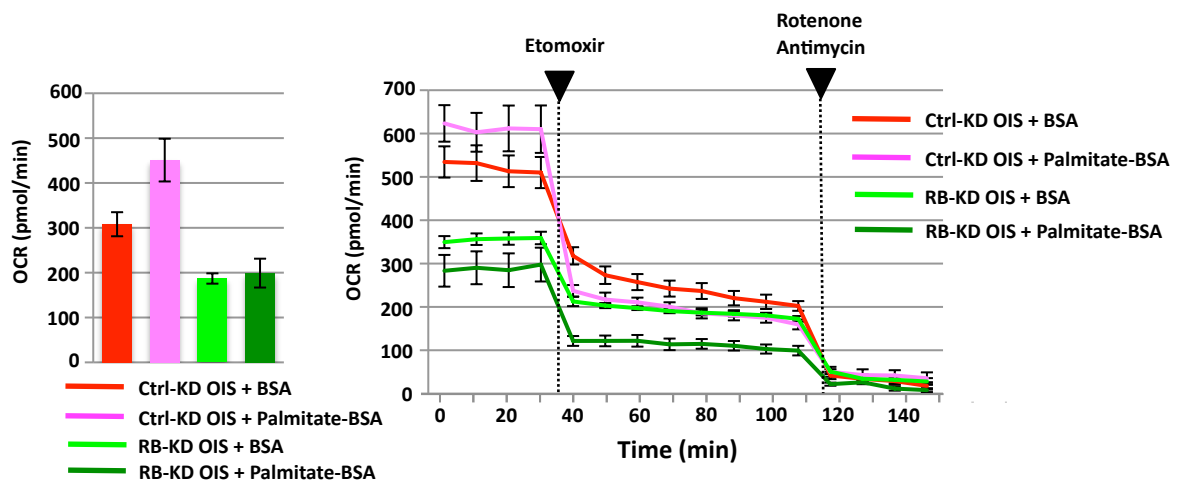

Fig. S8

Supplement: Supplementary file 1 [file acel0014-0689-sd1.pdf]
